# Attitudes of German General Practitioners Toward eHealth Apps for Dementia Risk Reduction: Qualitative Interview Study

**DOI:** 10.2196/56310

**Published:** 2025-01-22

**Authors:** Adrian Schultz, Melanie Luppa, Markus Bleckwenn, Steffi G Riedel-Heller, Andrea Zuelke

**Affiliations:** 1 Institute of Social Medicine, Occupational Health and Public Health Leipzig University Leipzig Germany; 2 Institute of General Practice Leipzig University Leipzig Germany

**Keywords:** eHealth, dementia, primary care, lifestyle, risk factor, older adults, prevention, brain health

## Abstract

**Background:**

eHealth interventions constitute a promising approach to disease prevention, particularly because of their ability to facilitate lifestyle changes. Although a rather recent development, eHealth interventions might be able to promote brain health and reduce dementia risk in older adults.

**Objective:**

This study aimed to explore the perspective of general practitioners (GPs) on the potentials and barriers of eHealth interventions for brain health. Understanding the perspective of GPs allows us to identify chances and challenges for implementing eHealth apps for dementia risk reduction.

**Methods:**

We conducted semistructured expert interviews with 9 GPs working in an outpatient setting in and near Leipzig, Germany. Data were fully transcribed and analyzed using a process model of qualitative content analysis with codes and categories being constructed inductively and deductively.

**Results:**

We found generally favorable but balanced views of eHealth apps for brain health. Eight themes were identified and elaborated on in the data as follows: “addressing dementia,” “knowledge about dementia,” “need for information,” “potential for prevention,” “chances for apps for prevention,” “development of apps for prevention,” and “barriers of apps for prevention.” GPs talked mostly about how and when to address dementia and the requirements for their use of eHealth apps for dementia prevention. GPs stated that they only addressed dementia once abnormalities were already present or less frequently when a patient or relative expressed a direct wish, while individual dementia risk or standardized diagnostic during routine check-ups were mentioned much less frequently. According to GPs, knowledge about dementia in patients was low; therefore, patients expressed little need for information on dementia risk factors and prevention in GP practices. Most patients wished for quick information regarding diagnostics, treatment options, and progression of the disease. GPs mentioned a lack of overview of the available eHealth apps and their content. They also expressed a fear of inducing health anxiety when talking to patients about risk factors and prevention.

**Conclusions:**

GPs want patients to receive relevant and individualized information. Prerequisites for the use of eHealth apps for dementia prevention were app characteristics related to design and content. GPs need to address dementia more routinely, assess relevant risk factors, and aid patients in a preventive role. Concerns were expressed over limited effectiveness, overwhelming patients, limited use in clinical practice, and only targeting patients with an already low risk of dementia.

## Introduction

### Background

Dementia is one of the most common neurodegenerative diseases. In Germany alone, dementia is one of the 5 most common diseases prevalent at the time of death [1]. At the same time, dementia is connected with a high burden of illness and excess costs of illness that are 2.5 times higher when compared to people without dementia [2]. Without available curative therapies, risk factors for dementia are a primary focus for dementia prevention. According to the latest report of the Lancet Commission on Dementia Prevention, Intervention, and Care, 45% of dementia cases can be explained by 14 modifiable risk factors (low levels of education, hearing impairment, hypertension, smoking, obesity, depression, physical inactivity, diabetes, higher alcohol consumption, traumatic brain injury, air pollution, social isolation, high levels of low-density lipoprotein cholesterol, and visual impairment [3]). This underlines the potential of dementia risk reduction by risk factor modification. In Germany, 38% of dementia cases are estimated to be due to 11 modifiable risk factors [4]. The use of eHealth apps has garnered considerable attention as a potential tool to assist in promoting brain health and mitigating dementia risk [5].

General practitioners (GPs) working in primary care usually consult their patients regularly and could potentially identify risk factors and early markers of dementia. GPs are also perceived to be an important support body with a high level of competence and trust [6] and play a central role in advising patients on health-related matters. Therefore, the primary care setting is crucial for the assessment of dementia risk factors and early dementia diagnosis. However, existing research has shown that dementia risk reduction is not routinely conducted in primary care settings [7,8]. Only few existing studies examined the views of GPs on dementia risk reduction.

While older adults are the most rapidly growing share of internet users in Germany [9], their use of eHealth tools has been found to be significantly lower than in other (European) countries [10]. The general use of health apps was low in a population-based sample of older (aged ≥60 years) German adults conducted in 2016, with only 16.5% reporting previous use of health apps. Concerns about data protection and lack of trust in eHealth tools were the most commonly named barriers [11]. Although these findings indicate a rather skeptical view of older German adults on eHealth tools, findings from the @ktiv-trial, testing an eHealth intervention for depression, found higher levels of adherence in older than in younger users, indicating the potential benefits of eHealth interventions for older adults [12].

Digital interventions aimed at enhancing brain health and mitigating dementia risk factors are currently an important focus of research [13-15], which explores potential benefits and challenges of digital apps for dementia risk reduction, highlighting their capacity to deliver personalized interventions and facilitate self-management on a large scale. However, despite these advancements in the field, only limited attention has been directed toward understanding the perspective of GPs, who play a critical role in adoption and implementation of these apps into clinical practice. Therefore, knowing the potentials and barriers before the development and implementation of such measures can be important to enhance potential. To the best of our knowledge, no study so far has examined the attitudes of German GPs toward eHealth apps for dementia risk reduction.

### This Study

Consequently, we conducted 9 qualitative expert interviews with German GPs practicing in the Leipzig area to gain insight into GPs’ subjective experience with older patients, how GPs address topics of dementia risk factors, and the possibilities of eHealth apps for prevention and managing dementia risk. Qualitative research can provide an understanding of subjective experiences, attitudes, and challenges GPs face. Qualitative studies can help explore and explain social relations between the health care system and the way patients and providers interact. In addition, qualitative approaches allow depicting aspects that might be lost by transformation of experiences into numerical forms for proper statistical analysis [16,17]. We aimed to identify possible chances and hindrances of eHealth apps for prevention of dementia and challenges for implementation. Furthermore, we aimed to provide specific recommendations toward goals, content, challenges, and possible implementation of eHealth apps to reduce dementia risk.

## Methods

### Study Design

This study uses a qualitative semistructured interview design. We conducted interviews with GPs via telephone using guided interviews. Telephone-based interviews were conducted to increase convenience and flexibility for participating GPs. Furthermore, we aimed to reduce the risk of social desirability bias and keep technical requirements for GPs to a minimum, arguing for telephone- instead of video-based interviews. Guidelines for the interview were designed to cover a prespecified topic list focused on dementia prevention, attitudes toward eHealth apps for dementia prevention, and technology-related factors. Although participants were reminded that the interview focuses on dementia prevention, GPs frequently steered the conversation toward identifying and managing dementia. This divergence from the intended focus of prevention was noted and addressed in real time by interviewers. Participants were encouraged to address further topics not covered by the interview guidelines at the end of the interview. We used a process model of qualitative content analysis as developed by Mayring [18] to identify key themes using inductive and deductive coding.

### Sample

Participants were GPs with practices in an outpatient setting (individual practice, joint practice, or medical center) in the city of Leipzig, Germany, and its surrounding region. No further inclusion or exclusion criteria were applied. Eligible GP practices were recruited from a database at the Institute of Social Medicine, Occupational Health and Public Health, including 53 GPs who had participated in previous studies or expressed interest in participating in future research. This sample contained GP practices from all regions of the city of Leipzig, comprising urban and suburban areas, as well as practices from the environs of Leipzig. All 53 GPs were contacted via telephone and email, of which 5 (9%) refused participation. Of the remaining 48 (91%) GPs, 12 (23%) expressed interest in participation. In 3 (6%) cases, interviews could not be scheduled due to time constraints on the side of GPs; therefore, the final sample included 9 (17%) GPs. On the basis of previous experiences conducting qualitative interviews, as well as narrowly defined research questions and a relatively homogeneous study sample [19,20], we expected to reach data saturation after 8 interviews. Interviews were conducted in a timeframe of roughly 25 minutes each. Participating GPs received detailed study information, an informed consent form, account data forms for the transfer of the expense allowance, and a sociodemographic questionnaire in advance via postal service. At the beginning of the interviews, interviewers informed participants about the method of expert interviews and encouraged them to share their experiences and opinions openly.

### Analysis

All interviews followed the same general structure. Before the interviews, participants were given a short introduction on the interview topic identical to the previously received detailed study information. Interviewers informed GPs that the goal of the study was to gather information on how informed older patients are about the risk and protective factors of dementia and their willingness to use eHealth apps to manage dementia risk. In addition, they were informed that there was an interview guideline and encouraged to share their own experiences and opinions. The interview guideline was developed for this study; a full English translation is provided in Multimedia Appendix 1.

First, interviewers asked GPs whether they address the topic of dementia and cognitive ability with their older patients. If so, they were further asked with which groups of patients they bring the topic up and whether older patients addressed dementia at appointments themselves. Second, GPs were asked to assess the knowledge of patients about dementia and respective risk or protective factors. In the third section of the interview, interviewers inquired if patients wished to receive information on dementia prevention. In the fourth section, interviewers gave a brief description of how modifiable risk factors, for example, managing cardiovascular risk, increasing physical and social activity, or optimizing diet, have a positive impact on cognitive ability. Interviewers then asked participants which risk factors they considered especially promising for dementia risk reduction, whether they bring up modifiable risk factors when talking about dementia with their patients, and which risk factors patients might already know. In the following section, participants were asked to assess the potential of eHealth apps for managing cognitive decline and reducing the risk of dementia. In the sixth section, participants were asked to imagine a respective eHealth tool that addressed modifiable risk factors (eg, diet, physical and social activity, cardiovascular risk) via standardized questionnaires and that allowed patients to receive individualized notifications and information on how to reduce their personal risk for dementia. Following this, GPs were asked to assess whether older patients would make use of this program, which patients they would recommend the program to, and whether they would recommend all of it to their own patients. In section 7, GPs were asked to elaborate on facilitators and barriers for such a program. In the last section, interviewers asked for the GP’s opinion on the implementation of respective eHealth apps for the prevention of dementia and which information they would need to assess the use of the service. Technical requirements, content, criteria of quality, measurable effect, privacy, and cost were given as anchors. Closing the interview, participants were thanked for their collaboration and asked whether they had an opinion, question, or if any topic was not covered in the interview.

All interviews were audio-recorded, fully transcribed and content-analyzed using MAXQDA 2022. We formed codes inductively and deductively according to the process model developed by Mayring [18] and the analytic pathway 1 as identified by Raskind et al [21] on qualitative data analysis practices in health education and health behavior research. Three trained scientific project members with experience in qualitative research independently coded and revised all interviews. The coding scheme was derived from an initial coding template based on literature, the interview guide (deductive coding), and emerging codes from the interview material (inductive coding, second-level coding within a domain, and openness for open coding during this stage). Methodological rigor was ensured by agreeing on a rule and process model-based approach before coding, iterative processing of reviewing coded data, discussing coded data in a group setting to develop and reach agreement on salient themes, repeated comparisons among coded data, combining similar or related codes into themes, and extracting themes from within codes [18,21]. After the final group discussion, categories were reviewed and given examples from the transcripts.

### Ethical Considerations

This study was performed in accordance with the principles of the Declaration of Helsinki in its revised version from 2000. The Ethics Committee of the Medical Faculty of Leipzig University, Germany, approved the study (587/21-ek). Participating GPs were informed of their right to withdraw from the study at any time without consequences and provided written informed consent before participation. Each participating GP was assigned an identification number and all data were deidentified for analyses. GPs received a reimbursement of €100 (US $103).

## Results

### Sample Characteristics

Table 1 shows the collected sociodemographic data and information for the GPs in the sample.

**Table 1:** Sample characteristics (N=9).

Characteristics		Values
**Sex, n (%)**		
	Male	4 (44)
	Female	5 (56)
Age (y), mean (SD; range)		46.3 (8.8); 33-57
**Duration of practice as resident (y), mean (SD)**		10.3 (5.5)
	11-25, n (%)	3 (33)
	26-50, n (%)	4 (44)
	51-75, n (%)	2 (22)
**Older patients treated per quarter, n (%)**		
	51-100	1 (11)
	101-500	4 (44)
	501-1000	3 (33)
	>1000	1 (11)
Estimated percentage of older patients with dementia, mean (SD)		20.11 (11.1)
**Form of practice, n (%)**		
	Individual practice	4 (44)
	Joint practice (*Gemeinschaftspraxis*)	3 (33)
	Medical center	2 (22)

### Themes and Subthemes Identified

We identified 8 themes, each including 2 to 12 subthemes. Table 2 shows the overall frequency of quotes per subcategory.

The theme mentioned most frequently was addressing dementia. On the level of subthemes, requirements for the implementation of eHealth apps were mentioned more frequently than other subthemes. GPs emphasized personal requirements for using an eHealth app for dementia prevention in their care. Themes and subthemes with respective key quotations are described in Multimedia Appendix 2.

**Table 2:** Frequency of themes (N=8) and subthemes (N=28).

Themes and subthemes			Frequency, n^a^
**Addressing dementia**			54
	Cause: abnormalities	17	
	Cause: high risk of dementia	1	
	Cause: routine checkup	3	
	Cause: affected relatives	3	
	Cause: initiative of patient or relatives	12	
	Expressed wishes toward GP^b^	18	
**Knowledge about dementia**			13
	High knowledge	2	
	Low knowledge	11	
**Need for information**			17
	For diagnostics, treatment options or progression	11	
	Psychosocial aspects	4	
	For risk factors	1	
	No need for information	1	
**Potential for risk reduction**			35
	Most promising risk factors	12	
	Addressing risk factors	11	
	Risk factors known by patients	9	
	Risk factors are disregarded	3	
**Chances for eHealth apps for dementia risk reduction**			21
	Advantages for patients	11	
	Advantages for practitioners	9	
	Low interest of patients	1	
**Development of eHealth apps for dementia risk reduction**			25
	Characteristics qualifying patients for apps for dementia risk reduction	13	
	Characteristics rendering patients unsuitable for eHealth apps for dementia risk reduction	10	
	Characteristics of eHealth apps for dementia risk reduction	2	
**Barriers to eHealth apps for dementia risk reduction**			17
	General barriers	3	
	Concerns	10	
	Limited use	4	
**Implementation of eHealth apps for dementia risk reduction**			33
	Prerequisite: accessibility	13	
	Prerequisites: characteristics of eHealth apps	10	
	Integration into GP care	10	

^a^Frequency of reference to the respective theme or subtheme in the total sample (n=9 GPs); multiple mentions of themes or subthemes per GP possible.

^b^GP: general practitioner.

### Addressing Dementia

GPs overwhelmingly addressed dementia in their care only when abnormalities were already present and noticeable by either the GP or their staff. Routine checkups or concerns of relatives played a minor role with only a singular mention of high dementia risk being the cause for addressing dementia in routine care:

So we address that routinely when doing check-ups, starting at 70. And in patients under 70 who have personal concerns or an increased risk of dementia from my perspective.

GP5

The initiative of patients or relatives was named frequently. GPs mentioned that patients rarely take initiative action regarding dementia risk reduction. Instead, GPs stated that relatives noticed changes in patients and referred them to GPs with a wish for diagnosis:

Oh well, when relatives register for consultation hours, they nearly always name somewhat of a reason, if it’s acute. And then they’ll say, “Dad is odd,” or “My husband is odd” And surely also “can’t we take a look?” and actively ask whether it’s the case [dementia]. So yes, it’s talked about quite directly.

GP2

Congruently, wishes expressed toward GPs had the most quotations within the theme. Wishes varied and ranged from explicit wishes for fast diagnosis to less specifically expressed emotional needs:

They [patients] surely want to hear that they are not forgetful when they are. Or can’t remember certain things. And whether there is medication. And get their fears off their chest, which they surely can’t articulate like that at home. Because it is a kind of weakness, how they feel about that. So this [GP practice] is a protected environment, where you can slowly move forward and ask whether it’s still normal. So they slowly advance, maybe talk about an occurrence.

GP4

### Knowledge About Dementia

All GPs described patients’ knowledge about dementia as low. These included symptoms of dementia, especially in contrast to age-related cognitive decline, mental disorders in general, as well as types and progression of dementia:

I think patients don’t know much about that [early signs of dementia]. Especially separating normal signs of aging and dementia is tough for a layman, I think. And in my experience, patients that worry a lot about having dementia more commonly suffer from senile depression or an anxiety disorder and not dementia. While the patients that do have dementia often negate that or trivialize that and think it’s normal when there’s surely an illness and a need for therapy, or at least help, from my perspective.

GP5

One GP mentioned that the level of education, personal experience, and degree of personal growth are relevant factors for high knowledge about dementia:

[Knowledge of elderly patients about dementia] is dependent on the level of education. It differs. Those who have the ability for differentiated thinking can assess that in a completely different way. And some confuse dementia with something else and simply say “Well, you’re surely demented” or something like that to their relative even though he isn’t. It’s not only related to the level of education but personal experience that they’ve made, with other patients. And to their own personality.

GP1

### Need for Information

In the experience of GPs, most patients want to receive information on personal diagnosis, treatment, and course of illness. Patients wish for standardized diagnostic as well as personally relevant assessments made by the GP. These assessments include personalized advice related to psychosocial factors, for example, living situation, relationships with family members, or concerns. Information regarding treatment options and the course of illness were mentioned more frequently than diagnostics. The need for information was only mentioned in regard to early diagnosis of dementia with only a singular mention of risk factors in the context of general health recommendations during check-ups:

For many it’s about the question: If I had dementia, how would I continue to lead my life? Can I stay independent? Do I need to change my living situation? What happens to my relatives or close ones, commonly spouses? That’s what I think concerns patients and is why dementia is omnipresent in our practice.

GP5

One GP elaborated that it is common for patients to have received misinformation about the use and limitations of pharmaceutical treatment for dementia by specialized health care providers. It is noteworthy that despite being included as an example in the interview guide and the inductive category system, no GP mentioned a need for information on the causes or risk factors of dementia:

If you suffer from dementia and see a psychiatrist, you get one or two different kinds of medication and that’s that. There is nothing about lifestyle, nothing about counseling the [social] surroundings, nothing with alternative therapeutic options, what is still possible. You get your pills prescribed and that was that.

GP6

### Potential for Dementia Risk Reduction

When asked about the most promising factors to be targeted for reducing dementia risk, GPs named social, cognitive, and physical activity. Cardiovascular risk factors were frequently named as part of routine care but are commonly not put into the context of dementia:

Measurable factors like blood sugar, blood pressure, and arteriosclerosis, that is definitely part of my job as a GP and an essential part of preventing dementia. The topic of social interactions, social contacts, exercise, I think those are very important. I talk about that, too, but I see my influence there as smaller because more initiative of the patient is required.

GP5

GPs stressed their concern over socially isolated older patients and their perceived limited influence on psychosocial factors (eg, social support). All participants stated that they addressed common risk factors in their practice but added that they are often unsure whether patients follow their recommendations. In the experience of GPs, patients often know about broad components of a healthy lifestyle and cardiovascular risk factors for illnesses, but not for dementia in particular:

[I do not mention risk factors] at every contact, but I especially use check-ups and so on to ask: how do you exercise. I always ask about alcohol, and, of course, their daily routine and such things. There, I often talk about what would be good to do more of and what to do less of. But if me addressing it [risk and protective factors for dementia] is enough, that is always hard to say.

GP8

One GP mentioned that they addressed lifestyle factors in general but did not give patients any recommendations regarding diet, due to limited potential for improvement:

[Regarding diet] I wouldn’t say that they [patients] should limit themselves or do less of anything. Disregarding alcohol, but nothing else. So, I wouldn’t say they need to up their vitamins, because of Vitamin C... I don’t see relevant potential there.

GP9

### Chances for eHealth Apps for Dementia Risk Reduction

GPs perceived eHealth apps for dementia risk reduction mostly as a chance to standardize patient education and integrate social factors into care. Strengthening self-efficacy and motivating patients was another point mentioned by participating GPs:

It really is mostly about relatives who would be very interested in helping their spouse, for example. Or help their parents. And then, of course, if a patient isn’t able to educate themselves about this application or read everything, with the help of relatives it would be very appreciated. I think that makes sense.

GP3

One GP mentioned the ability to self-administer screening tests before appointments as a chance to improve care:

[I would use it] just how I recommend a good depression scale if I know one, check yourself for depression. There it says a bit more, for example, moderate depression, and then I would offer to check with me again.

GP9

### Development of eHealth Apps for Dementia Risk Reduction

GPs characterized suitable patients for eHealth apps for prevention as high in health literacy and self-efficacy. Especially younger patients and patients with high media literacy were named as suitable for eHealth apps for dementia risk reduction. Accordingly, patients who would be unsuitable for such measures were characterized as older individuals or those who lived alone without help:

But the older people who are alone, I don’t think they could manage to do that. If I imagine two 85 year old seniors here, no. And one of those has dementia... They rather have questions like, how do I turn on the phone? And then getting to an application, that is really hard.

GP6

### Barriers to eHealth Apps for Dementia Risk Reduction

GPs mentioned they were worried that early preventive measures for at-risk patients could increase anxiety and worries about dementia in patients. They also argued that patients who would be likely to use these measures are both already overly anxious and therefore already active. This perception would limit the effectiveness and likeliness of use for GPs severely.

One GP elaborated that it would be important for them to use eHealth only for supporting already existing care and explicitly communicate barriers of eHealth and limitations of prevention for dementia. As an example, this GP listed individualized recommendations for lifestyle changes from an eHealth app.

### Implementation of eHealth apps for Dementia Risk Reduction

To implement eHealth apps for dementia risk reduction into their care, GPs mentioned that they would need to have an overview of the information provided by the app to administer its use. Furthermore, GPs emphasized a high degree of accessibility in terms of ease of use and low complexity. Reliable privacy policies and free use were mentioned as key factors for recommending such measures to patients. Most GPs advocated supervised use of preventive measures by qualified personnel (eg, GPs, psychologists, and nurses):

I imagine a certain type of older person at their computer, entering their data and the application says: “you have moderately severe dementia.” And I don’t want to imagine that patient alone with their computer.

GP5

Further ideas of GPs were integrating recommendations for eHealth apps for prevention into routine check-ups or cooperating with nursing services that can assess the need for lifestyle changes. Finally, one GP mentioned a lack of information regarding existing preventive measures for GPs and wished for vocational training, published guidance, and personalized letters or emails.

## Discussion

### Principal Findings

To the best of our knowledge, our study is the first to use a qualitative approach for examining GPs’ views on eHealth apps for dementia risk reduction, interviewing 9 German GPs working in an outpatient setting. Overall, GPs named more positive than negative aspects of eHealth apps for dementia risk reduction and displayed a balanced but positive attitude toward them. It is noteworthy that participants frequently steered the conversation toward early dementia diagnosis or dementia care management, although the questions focused on dementia prevention. This was also reflected in the coding system, as GPs frequently noted that they only addressed dementia when abnormalities were present and that patients’ need for information concerns only diagnostic and treatment options. Existing research has established that GPs are reluctant to perform early dementia diagnosis due to uncertainty, which seems to extend to dementia prevention [22]. Our findings may aid in the development and implementation of eHealth apps for dementia prevention and to inform discussions about acceptance and attitudes of eHealth apps for dementia risk reduction with primary care givers in Germany.

According to the typology of eHealth implementation theories developed by Heinsch et al [23], respective theories can fall into the 5 categories of centering agency, structure, relations, meaning, and norms. Agency-centered theories focus on an individual’s actions, beliefs, and attitudes as opposed to other theories that center, for example, on organizational environments or interactional processes. The topics covered by our participants are highly compatible with agency-centered theories, as there was no mention of structural, organizational, or other factors. Particularly, the Technology Acceptance Model (TAM) and the Unified Theory of Acceptance and Use of Technology (UTAUT) were reflected in our coding system. TAM proposes that technology acceptance and use are affected by perceived ease of use, perceived usefulness, and subjective norms, all of which were most frequently mentioned by our participants. UTAUT proposes that the intention to use eHealth apps is affected by effort expectancy, performance expectancy, social influence, facilitating conditions, and habit [23]. Effort expectancy and performance expectancy were factors GPs talked about in-depth, while social, environmental, and habitual factors only played a minor role, for example, for practical implementation purposes, such as delegating the use of eHealth app to staff. Addressing dementia due to high risk or during routine checkups was only mentioned 4 times. Therefore, many GPs seem to show little to no regard for primary prevention and dementia risk reduction, which is in line with recent findings from the Netherlands [6] and the United Kingdom [8], where GPs stated that they did not address dementia prevention or risk reduction with their patients in routine care.

According to the interviewed GPs, patients want to receive individualized information and apps they can work at their tempo. The social environment and relatives should be included in the use of the app. In addition, apps should be designed sensitively so as to not cause or reinforce worries regarding dementia and give realistic goals and expectations.

There seems to be a need for information on the part of patients, as GPs predominantly perceived their patients’ knowledge about dementia as low. This indicates potential for increasing knowledge, for example, via eHealth apps for dementia risk reduction. Patients seem to have no interest in information on prevention of dementia, and risk factors can be attributed either to no interest in these aspects or, in our eyes more likely, to no knowledge about modifiable dementia risk factors [14,24]. When patients are unaware of a link between lifestyle and dementia risk, they have no reason to seek out their GP for information.

In an earlier study, older patients expressed the wish for eHealth services to be integrated into usual GP treatment and had favorable opinions toward eHealth services when they supplemented care [25]. Congruently, GPs were generally also in favor of individualized risk assessment and lifestyle recommendations. In a recent study [26], only a small number of interviewed GPs saw self-monitoring as a useful area for implementation of eHealth apps for prevention measures.

In accordance with these findings, the use of eHealth apps for dementia risk reduction was also only addressed by one GP in our study, while the use for psychosocial care was generally favored. In existing research on the perception of internet-based and digital health apps in general, physicians see a variety of advantages and potential uses, particularly for preventive measures and lifestyle modifications [26-29]. A meta-analysis reported small to medium effect sizes for eHealth interventions for brain health on subjective and cognitive performance as well as risk profiles for dementia [13]. More recent studies showed beneficial intervention effects on risk factors for dementia in middle aged and older adults [30,31]. Communicating respective findings regarding eHealth interventions for dementia risk reduction to GPs might increase practitioners’ interest and convince them of their potential effectiveness. Against this background, raising awareness for the potential of dementia risk reduction and the role that eHealth interventions might play in this regard among GPs seems necessary to maximize the potential of risk reduction strategies.

Tying in with earlier studies [26], GPs stressed free access and ease of use. In addition, GPs felt unsure navigating the variety of digital health apps and wished for scientifically evaluated effectiveness to find appropriate apps. Despite the currently explorative stage of research, our results fit well into existing studies and allow us to draw narrow conclusions about the preventive use of internet-based or digital health apps for dementia risk reduction. A previous review identified wrongful data use, cost, and lack of quality of care as topics that concern patients the most regarding digital health apps and this fear seems to be warranted [32]. Therefore, potential eHealth apps for dementia risk reduction should prioritize no costs for patients in their financial planning if they want to ensure maximum use by GPs and patients alike. Similar to the studies that attribute the low use of digital health apps in practice to concerns regarding safety, interviewed GPs in our study also frequently mentioned security concerns and privacy issues. The most frequently named concerns in our study (limited use, low effects, and inducing anxiety and worries) can be explained both within the theoretical frameworks of TAM and UTAUT, as they seem to reflect the factors of perceived usefulness (TAM) and expected performance (UTAUT). A recent study with a similar design to our study from the United Kingdom also found that the fear of inducing health anxiety seems to be common in GPs regarding dementia prevention [8].

Another perceived barrier not mentioned in previous studies was that eHealth apps for prevention measures would only be used by patients with (1) high anxieties and worries regarding dementia, (2) high self-efficacy, or (3) high health literacy, thereby limiting potential. Generally, existing research suggests that digital health interventions improve health literacy [33,34]. However, the use of digital health apps is affected by structural inequalities, such as low socioeconomic status, ethnicity, and education status, which contribute to disparities in health literacy as well as use of additional health education programs [35]. In contrast, eHealth interventions can use various channels to provide information, for example, audiovisual or interactive formats, which may facilitate the inclusion of older adults with lower levels of education or health literacy [36]. This view was also expressed by one of the GPs in our study (GP8). Previous studies indicate that the effects of digital health app use on health behaviors could be mediated by self-efficacy [37], which is a different effect than the one frequently proposed by our participating GPs (eHealth apps increase knowledge for already motivated patients) and could be communicated to GPs interested in the mechanisms of future internet-based preventive measures. An earlier study focusing on older adults in a low socioeconomic community found increased computer health literacy and self-efficacy because of a targeted computer intervention [38]. The GPs in our interviews also focused on increased self-efficacy and participation of patients and their social environments as one of the most promising factors for the use of eHealth apps for prevention measures. The barriers discussed by our participants and the concerns over implementation into routine care seem to reflect a fear of a lower quality of care or a loss of trust between the physician and the patient when relying too heavily on digital apps. The review by Ramachandran et al [39] on the impact of eHealth on relationships and trust in primary care shows that eHealth apps can even increase trust when patients perceive that they are receiving personalized and collaborative care. This indicates that GPs could be more in favor of eHealth apps for dementia prevention when used in a hybrid care model, where GPs use eHealth prevention tools when assessing high dementia risk in a patient. Our results allow a potential investigation of the role of eHealth apps that GPs would prefer, although the integration into routine care is a longstanding debate with many more factors to consider [40-42]. It would be interesting to see future evaluations of eHealth apps for prevention measures include constructs, such as self-efficacy, perceived usefulness, physician-patient trust, and measurement of possible covariates that might influence the effect of eHealth apps for prevention measures. Research based on social cognitive theory has consistently found that self-efficacy facilitates a change and uptake of change in health behaviors [43] and is, therefore, a relevant outcome for evaluative studies that test digital health apps.

GPs mentioned both a lack of overview of existing apps and their respective content and were unsure about how to integrate them into their clinical practice. Therefore, eHealth apps should be designed in a way that frequently refers to the GP in a primary care setting, for example, via having tips that direct patients to their GP. Furthermore, referral to eHealth apps by trusted sources like GPs has been suggested as a promising approach to increase older adults’ interest in eHealth tools [35]. This could serve to strengthen the role of the GP in providing care and reduce unrealistic expectations of patients regarding the possibilities of the app, reducing 2 important barriers at once. To maximize cooperation with GPs, internet-based prevention measures should serve a clear and identifiable role in the care of patients, for example, as part of comprehensive psychosocial care provided by the GP or part of self-monitoring in-between appointments. Developers of eHealth interventions aimed at brain health and dementia risk reduction might benefit from cooperation with GPs early on in the process of designing interventions. Furthermore, offering concise information for GPs on the device, that is, contents, target group, administration in GP practice, and tutorials for GP practice could further enhance acceptance of eHealth tools among GPs and strengthen cooperation between researchers, developers, and practitioners toward the goal of dementia risk reduction [4]. A possible application for eHealth interventions for dementia risk reduction and other digital health apps should further focus on supplementing in-person health care to increase health literacy and mend care in areas with weak health infrastructure or particularly hard to reach patients [34,38,45,46]. Textbox 1 summarizes recommendations for the development and implementation of eHealth tools for dementia risk reduction, as expressed by participating GPs.

**Recommendations for design and implementation of eHealth tools for dementia risk reduction.**

- Ease of use, low complexity: relevant information is readily traceable within the eHealth tool, intuitive navigation
- Individualized recommendations: provide guidance and recommendations based on individual dementia risk profiles and stage of motivation for behavior change
- Sincerity about the potential for dementia prevention: avoid overemphasizing individual means of risk reduction (eg, “risk reduction” instead of “prevention”)
- Inclusion of partners and relatives: provide means for partners or other relatives to interact with the eHealth tool, for example, to coach patients in using the app
- Privacy and data protection: ensure privacy of user data, prohibit wrongful transfer of participant data to third parties
- Accessibility: provide the eHealth tool free of cost, easily findable in app stores
- Inclusion of general practitioners (GPs) and other health professionals: provide structured information on available eHealth tools for dementia risk reduction, including evidence of effectiveness, for GPs. Train health professionals (GPs, medical assistants, nurses, psychologists, etc) in the use of the app to facilitate supervision of patients’ use of eHealth tools

### Discussion of Methods

Qualitative research does not allow direct comparability via quantified scores and readily defined constructs. However, this tradeoff allows a more accurate depiction of ambivalences, subtleties, and possibly contradicting opinions that would be lost if focusing on rigorously established scales. Therefore, we decided on a structured, but explorative design to gain as broad of an insight as possible. Using the qualitative research approach of Mayring [18] in formulating the interview guide and data analysis with a very high degree of structure, validity, reliability, and generalizability, we hope to provide robust information. This procedure seems particularly appropriate, given the novelty of eHealth apps for dementia risk reduction.

### Strengths and Limitations

Our study assessed a timely topic for means of dementia risk reduction, that is, the use of eHealth apps. GPs can play a major role in providing recommendations for lifestyle changes to reduce dementia risk. Therefore, assessing GPs’ attitudes toward eHealth for dementia risk reduction may likely provide useful evidence for both the design and implementation of apps, as well as uptake and acceptance by patients. Still, certain limitations need to be considered when interpreting our findings. Although the interviews focused on primary prevention, that is, dementia risk reduction, several GPs referred to the use of eHealth for secondary prevention in our study. Furthermore, heterogeneity in participating GPs’ age may have influenced our findings. It might be possible that younger participants may generally be more familiar with eHealth tools, while GPs in their 50s might be less accustomed to apps, possibly rendering them more skeptical toward eHealth for dementia risk reduction. To ensure the timely execution of interviews and due to time constraints of the project, GPs were recruited from established networks of GPs, raising the risk of selection bias and resulting in a rather homogeneous sample. Because we aimed to keep effort for GPs to a minimum, we only assessed limited sociodemographics and further information about GPs. A larger, more diverse sample recruited across various channels may improve the generalizability of findings in future studies. However, this study aimed to provide a first insight into German GPs’ attitudes toward eHealth for a fairly new topic, that is, dementia risk reduction. We are confident that our findings provide valuable insights that can inform larger-scale surveys on the topic as well as the design of eHealth interventions aimed at reducing dementia risk.

### Implications and Recommendations for Future Research

Future eHealth interventions for brain health could be developed and implemented as part of a blended care approach, similar to strategies already used in psychiatry and primary care [47-49]. Considering the intended form of use (eg, directly supervised, supported use in between appointments) before developing the app and its features would allow increased performance expectations and better evaluation. As lifestyle interventions aim to induce behavioral change, future studies for developing and evaluating eHealth apps for brain health should include measures that can assess the constructs that are intended to induce this behavioral change alongside relevant outcome measures. Along with general efficacy and effectiveness evaluations, this could allow a better understanding of not just if eHealth apps for prevention measures work but how they work, as few available interventions include comparable constructs (eg, changing health beliefs, increasing self-efficacy, and introducing or improving existing health behavior). A proof-of-concept study evaluating the Dutch MijnBreincoach app indicated no change in beliefs and attitudes despite positive evaluations [50], whereas in another study, the same app improved adherence to the Mediterranean diet, BMI, and knowledge of dementia risk reduction [30]. We recommend that future researchers rely on unified theory-based questionnaires for health beliefs, such as the Motivation to Change Lifestyle and Health Behaviors for Dementia Risk Reduction scale [51].

We further propose that researchers and developers consider the needs and concerns of GPs named in this study and similar studies to increase acceptance and adoption by practitioners. Similarly, the factors influencing user acceptance should be assessed to make future interventions valuable to practitioners and patients alike. The development of future interventions should prioritize tailoring and personalization of eHealth apps for dementia risk reduction. Features, recommendations, and information given should be relevant to the individual patient and provide a positive approach toward change of lifestyle factors, without raising or increasing fear of dementia in users. Finally, future research should pay attention to general health inequalities and access to eHealth apps for dementia risk reduction [34,35,46]. Understanding existing disparities in access, use, and outcomes among populations with different socioeconomic backgrounds can allow equitable distribution and ensure that interventions reach those who need them.

## Appendix

Multimedia Appendix 1 Interview guide.

Multimedia Appendix 2 Themes, subthemes, and quotations.

## Abbreviations

GP: general practitioner
TAM: Technology Acceptance Model
UTAUT: Unified Theory of Acceptance and Use of Technology

## Data Availability

The datasets generated during and analyzed during this study are available from the corresponding author on reasonable request.
