# Supplementary material for: Attitudes of German General Practitioners Toward eHealth Apps for Dementia Risk Reduction: Qualitative Interview Study
Source: JMIR Form Res. 2025 Jan 22;9:e56310. doi: 10.2196/56310 (PMC11799816; doi:10.2196/56310)
Supplement: Multimedia Appendix 1 [file formative_v9i1e56310_app1.docx]

**Multimedia Appendix 1**

**Schultz, A., Luppa, M., Bleckwenn, M., Riedel-Heller, S.G., Zülke, A.E.:** Attitudes of German General Practitioners Toward eHealth Applications for Dementia Risk Reduction: Qualitative Interview Study

**Interview guide**

Addressing dementia in primary care

Do you address the topic of dementia or cognitive performance when dealing with your older patients?

- If yes, with which groups of patients?

Do your older patients bring up the topic of dementia in your practice?

- If yes, what requests or wishes do they express regarding this?
- If yes, do they bring it up in front of their general practitioner? In front of the practice staff?

Patients‘ knowledge about dementia

How would you rate your older patients‘ knowledge about dementia?

Patients‘ wishes for information about dementia

Do your patients express a desire for information or education about dementia and risk factors for the condition?

If yes, how do you address this request? (e.g., through a conversation, providing informational materials, referring to specialized societies or other sources of information, or referring to neurological or psychiatric specialists?)

Potential of dementia risk reduction

The research on possibilities of dementia risk reduction has made significant progress in recent years. There is evidence for numerous modifiable risk factors, such as the management of cardiovascular risk factors, increasing physical and social activity, or optimizing nutrition, which can have positive effects on cognitive performance.

- Which modifiable risk factors do you consider particularly promising for the prevention of cognitive decline and dementias?
- Do you address these modifiable risk factors with your older patients?
- What do you think about the level of awareness of these risk factors among your patients?

Potential of internet-based tools for dementia risk reduction

More and more older people are using the internet today to gather information, including health-related topics. This development could also bring opportunities for preventive approaches against cognitive decline and dementias.

- Are you familiar with internet-based information resources for dementia-related issues?
- What advantages or opportunities do you see in using the internet for your patients? (e.g., usability independent of time and location, increasing health literacy, empowerment, motivating behavior change, etc.)
- Do you see potential benefits or advantages for yourself as a healthcare provider?
- In which patients would the use of online resources be suitable?
- In which patients would the use of online resources not be suitable?

Development of internet-based tools for dementia risk reduction

Assuming there was an internet-based program, such as a website or an app, designed to help patients assess and manage their individual dementia risk. This program would use standardized questions to capture modifiable risk factors (e.g., dietary habits, physical and social activity, cardiovascular risk factors) and provide personalized tips for risk management. With this program, patients could receive tailored advice and information to benefit their cognitive performance.

- Do you think older patients would use such a tool?
- For which patients would the use of such a program be suitable?
- For which patients would the use of such a program not be suitable?
- Would you recommend such an online tool to your patients?

Limitations and barriers

What disadvantages or limitations do you see in the use of online tools for dementia risk reduction?

- from the perspective of your patients?
- for yourself as a healthcare provider?

Implementation

Imagine you wanted to recommend an online program for dementia risk reduction to your patients - what framework and information would be important for you to make this decision? For example...

- technical requirements?
- contents of the tool/target audience?
- quality criteria/effectiveness?
- data privacy and security?
- costs?

From the perspective of your patients, what specific measures or aids do you think would be necessary to enable the use of such an online tool in healthcare?

Open issues

Have I missed anything from your perspective? Is there anything else important that you would like to mention?

End of interview

Thank you very much for taking the time for this interview! By doing so, you are supporting our research on possibilities for dementia prevention and making an important contribution to the care of older patients.
