# Supplementary material for: Attitudes of German General Practitioners Toward eHealth Apps for Dementia Risk Reduction: Qualitative Interview Study
Source: JMIR Form Res. 2025 Jan 22;9:e56310. doi: 10.2196/56310 (PMC11799816; doi:10.2196/56310)
Supplement: Multimedia Appendix 2 [file formative_v9i1e56310_app2.docx]

**Multimedia Appendix 2**

**Schultz, A., Luppa, M., Bleckwenn, M., Riedel-Heller, S.G., Zülke, A.E.:** Attitudes of German General Practitioners Toward eHealth Applications for Dementia Risk Reduction: Qualitative Interview Study

Themes, subthemes, and quotations.

| Themes and subthemes | | | Quotations |
| --- | --- | --- | --- |
| **Theme 1: addressing dementia** | | | |
|  | ST^a^ 1: cause: abnormalities | | “In the context (management of diabetes), the topic of dementia is often addressed of course, when we notice that patients also can’t deal with their medication, when we notice that problems exist, for example when patients use insulin regimes, when measuring blood sugar levels that patients have to perform on their own at home. When that happens, it’s addressed very actively. But not primarily that we call it dementia, but that we address it via different kinds of questions, how they get along with the measuring equipment, injections, whether and how they deal with their insulin regiments when they get their medication plan, whether they understand it. Of course, there is the geriatric assessment and such, which we conduct regularly. So addressing dementia by talking about it to everyone who comes in here to measure their blood pressure, not so much. But when married couples come in, then you are fairly quickly asked about bladder issues and this and that. And through these workarounds it's more likely for me to address dementia. Other than that, what patients often complain about is maybe the start of slight cognitive deficits, you know? And people have that most likely as they age. And that's about everything.” [GP7] |
|  | ST 2: cause: high risk of dementia | | “So, we address dementia routinely when conducting health check-ups from 70 years onwards. And with patients who are under 70 and have personal worries or, in my view, have an increased risk of dementia.” [GP5] |
|  | ST 3: cause: routine checkup | | “In principle, we try to talk very openly about dementia. Definitely at the check-ups. And at a certain age, we conduct geriatric assessments. We have different tests we conduct.” [GP5] |
|  | ST 4: cause: affected relatives | | “When the patient has personal concerns because of that. For example, they just came into contact with dementia. So, because they saw dementia in someone else, you sometimes have to answer questions regarding the illness or prevention.” [GP1] |
|  | ST 5: cause: initiative of patient or relatives | | “Some patients address that very actively. What is relatively common is that a spouse addresses something, when something is noticeable. Or children. That is relatively common. But sometimes there are patients that mention forgetfulness or similar symptoms themselves. Yes.” [GP7] |
|  | ST 6: expressed wishes toward GPs | | “So yes, some do have wishes [...]. But most, I feel like, just want to address it and don't really have an idea on how to proceed after that. It's more like they rely on what we say or whether we say that it's not that bad. There are some patients who say ‘I’d like to see a neurologist’ Someone expressing that they want to go to a psychiatrist because of that, that doesn't happen.” [GP8] |
| **Theme 2: knowledge about dementia** | | | |
|  | ST 1: high knowledge | | “Generally, yes. But that not only has to do with level of education, but personal experience that you made yourself, or with other patients. And with your own personality. If someone can stand above things and is very kind with their relative, that person can amass knowledge about dementia and cope with it in a wholly different way than someone who doesn't want to get things too close to them.” [GP1] |
|  | ST 2: low knowledge | | “I think knowledge isn't huge. I mean, most know that there is such a disease and, of course, have examples in their surroundings, but how disease onset can look like and course and what would be important to do then, I'd say, there is no huge knowledge there, no. There is dementia and it mostly shows in forgetfulness, but specific facts, I think I'd say no.” (GP8) |
| **Theme 3: need for information** | | | |
|  | ST 1: for diagnostics, treatment options/progression | | “I think, he (the patient) wants specific information that concern himself. He doesn't want to hear what he can read on Wikipedia or knows from his neighbor, but wants me, as his GP, to put his personal situation in the context of dementia. So either the question ‘If I do have dementia, what can we-, what does that mean for me? What can we change about that? How can we deal with that?.’ Or rather the question, regarding prevention of dementia ‘What can I do specifically in my situation?’ And in that situation, I believe he doesn't want to hear blank statements like, I don't know, ‘be among people and keep your social contacts.’ Instead, he wants me to reflect his environment or his situation and give him an individual recommendation. That is my personal impression.” [GP5] |
|  | ST 2: psychosocial aspects | | “Exactly, for example the situation: ‘Mrs. Müller, for you, after the stroke you had ten years ago, it’s a different situation.’ Or ‘Mrs. Maier, I supervised your mother in the nursing home, and for her, the course [of disease] looked like this or that. With you, we can do this and that to alter the course.’ Or, ‘Mrs. Schultz, you are rather burdened by caring for your chronically ill husband, that possibly plays a part in how you experience your own sickness and development [of disease].’ So that, I think, is what people expect when seeing their GP. Solely providing information, providing facts, I think patients do not expect that and, frankly, I don't provide them with that, this purely factual knowledge.” [GP5]  “If you suffer from dementia and see a psychiatrist, you get one or two different kinds of medication and that’s that. There is nothing about lifestyle, nothing about counseling the (social) surroundings, nothing with alternative therapeutic options, what is still possible. You get your pills and that was it.” [GP6] |
|  | ST 3: for risk factors | | “So for risk factors—I will tell it like this, if we talk about it, it’s mostly a little too late. Sometimes it comes up when doing check-ups in elderly patients, then it’s like: you tell them to exercise a lot, keep fit and keep in touch with contacts and so on, that you give general recommendations. But I’d say that’s a minority.” [GP8] |
|  | ST 4: no need for information | | “No, actually not. No, come to think of it, maybe once or twice a year. So it’s absolutely rare (for patients to ask for information on dementia or respective risk factors).” [GP9] |
| **Theme 4: potential for risk reduction** | | | |
|  | ST 1: most promising risk factors | | “People who have been active, including physically and mentally, they do have a kind of preventive effect. You notice that clearly. People who still read a lot, do crosswords, sodukos, and something like that, with them you feel like they are training their brains. But also, with physical activity—those who are outside and active a lot, with them you feel like they have a kind of preventive effect. With those cardiovascular things—yes, it’s certainly true, there is certainly evidence (for them to be an important risk factor for dementia). But I do have to say, honestly, with my patients I cannot really make a connection there, that for example someone who had three heart attacks already also had higher risk for dementia or something. [...]. Well, there is a little where I would say, that is certainly true and perfectly verifiable. But the activity factor, physically and mentally, probably plays an even bigger part.” [GP2] |
|  | ST 2: addressing risk factors | | “Yes, not always and at every contact, but especially at check-ups or something like that, I use the time to also ask: how physically active are you. I always ask about alcohol and, of course, daily routines and things like that, but—I often talk about that: it would be nice if you did a little bit of this and little bit less of that. But if addressing that alone is enough, is always hard.” [GP8] |
|  | ST 3: risk factors known by patients | | “In most cases, the heart diseases. So, everything concerning cardiovascular risk. That is well-known among patients. Integration into the social environment, I think that is not really in the minds of patients. Many of them, so to speak, get comfortable in their own four walls and live in their quote-unquote solitude.” [GP7] |
|  | ST 4: risk factors are disregarded | | “So, I wouldn't say they (patients) should restrict themselves in any way or do less of something, excluding alcohol of course, but nothing further. So, I wouldn't say they have to raise their vitamins, because vitamin C... I don't see relevant potential there. With cardiovascular risks, that’s my job anyway.” [GP9] |
| **Theme 5: chances of eHealth for dementia risk reduction** | | | |
|  | ST 1: advantages for patients | | “It’s mostly about the relatives (of patients) who would be very interested in helping their spouse for example. Or help their parents. And then certainly, when a patient is unable to get familiar with a program or read everything, to do so with the help of relatives, I think that would be appreciated. I think that makes sense.” [GP3] |
|  | ST 2: advantages for practitioners | | “We all know that patients sitting across from us only take in a fraction of what we say. And if I start talking about five risk factors, they might remember one. But I still do it and might hand something out to patients. Whether it’s informational material on paper or advice for a web page to say ‘take a look in peace and if you have any questions, we’ll talk about them next time.’ Or I ask them specifically, ‘have you looked at that, was there something that made sense to you, that you can think about, read or listen to at your own pace, in your own environment.’ Internet-based also uses video a lot, reading is not everyone’s first choice. And maybe also read about things that you (GP) did not mention yourself, yes.” [GP8] |
|  | ST 3: low interest of patients | | “When health applications came for different topics, I thought to myself: ‘That’s amazing.’ And now I'm surprised how little people want to use these. Even with depression, where everyone in his own little chamber can use it and be glad he doesn't have to see a therapist. Which is why they often agree to something like that. In the end, it’s used very little.” [GP1] |
| **Theme 6: development of eHealth applications for dementia risk reduction** | | | |
|  | ST 1: characteristics qualifying patients for applications for dementia risk reduction | “But I think the elderly that should be cared for with prevention are those who are going out for dancing or hiking groups anyway. Or meet up for coffee or come into contact with other people and do some activity. Or do rehabilitation sports and so on.” [GP1] | |
|  | ST 2: characteristics rendering patients unsuitable for eHealth applications for dementia risk reduction | | “The patients that are likely living alone. And are living alone and have no help regarding that (online-based preventive measures). That might not be able to deal with the internet. So, for these kinds of patients I’d say it’s not suitable.” [GP3] |
|  | ST 3: characteristics of eHealth applications for dementia risk reduction | | “I’d see it like this—I do not know any patient who on the internet, I would not particularly advocate engage with something like that (eHealth applications for prevention). Personally, I would not send my patient into automated programs. I would rather send them to ergo therapy, where patients get an individualized plan. But not anonymously for something like that.” [GP9] |
| **Theme 7: barriers toward eHealth for dementia risk reduction** | | | |
|  | ST 1: general barriers | | “So I think that the decisive factor is the personal experience people have with dementia. […] And that [experience as a starting point] is why you deal with the topic at all. So I don't think that factual knowledge or conveying facts and numbers influences people’s behavior.” [GP5] |
|  | ST 2: concerns | | “I think they (the patients) come in too late. Or for the relatives. It’s often too late there as well. They (relatives) cannot do that anymore (use online-services for dementia). Certainly, for some who are overly worried, who see a forgotten key as a criterion for dementia. There, it’s certainly not bad. Those are often the kind of people who are active anyhow in some way. Who take care at the right time. And if the child fell into the well already, then you would need to counsel the relatives. And support them somehow.” [GP4] |
|  | ST 3: limited use | | “Now as a GP, I am convinced about my role and I think it (implementation of eHealth for brain health) would not be possible without GPs. Ideally, I would wish that such an offer existed and the GP knows the respective offer.” [GP5] |
| **Theme 8: implementation of eHealth for dementia risk reduction** | | | |
|  | ST 1: prerequisite: accessibility | | “So, ease of use, simple questions, simple workflow. Where you can say: I don't have to navigate 50 modules and search for the things relevant to me. But there is a relatively stringent plan, that can be individualized for me, but coming more from the background. In a situation where someone who has handicaps visible in the mini mental state or so can use it differently than someone who has no clinically apparent symptoms and is maybe 65, active in life. Something that can be adapted to age, respectively, to their level. So yes, ease of use and a clear structure, a clear construction. That’s what I think is important.” [GP2]  “Costs, of course. It would leave me with a bad feeling if it would cost something. I would not find that very good. Gladly if it does not cost anything. I would not recommend it otherwise, no.” [GP4] |
|  | ST 2: prerequisite: characteristics of eHealth applications | | “(The formerly named aspects) would be important, but most of all the evidence. [Interviewer: That the effectiveness is proven?] GP: Yes. Or else it would be another toy that's paid for by insurance. What patients could surely pay on their own or get on their own.” [GP1] |
|  | ST 3: integration into GP care | | “So, can I delegate that (instructing patients on the use of eHealth tools) to someone else? Can a doctor in training do it? Can a student explain it or do I have to as a GP, because I have to explicitly bring myself into that matrix of thinking to teach it to the patient? So how easy is it to use—that is really decisive, because the easier it is to put it into practice for the entire staff, the more feasible the implementation.” [GP7] |

^a^ST: subtheme; GP: general practitioner
